# Effects of an Interaction and Cognitive Engagement-Based Blended Teaching on Obstetric and Gynecology Nursing Course

**DOI:** 10.3390/ijerph19127472

**Published:** 2022-06-18

**Authors:** Jiayuan Zhang, Yuqiu Zhou, Yingli Li

**Affiliations:** 1 Department of Nursing, Harbin Medical University, Harbin 163319, China; 2021022257@hrbmu.edu.cn (J.Z.); hlxzyq@126.com (Y.Z.); 2 Department of Medicine, Jiaxing University, Jiaxing 314001, China

**Keywords:** blended teaching, obstetrics and gynecology, nursing, cognitive

## Abstract

An interaction and cognitive engagement-based blended teaching mode was applied to obstetrics and gynecology nursing course to examine the effects on nursing students’ competency, self-directed learning level. A randomized controlled trail design was designed. The experimental group engaged with the blended teaching, and the control group was assigned a usual teaching. The level of competency, self-directed learning was compared between two groups. The total score and scores of each dimension of core competence and self-induced learning ability in intervention group were all higher than those in control group (*p* < 0.05).

## 1. Introduction

With the continuous reform and innovation of nursing education in China, it has become an inevitable trend to cultivate the comprehensive ability of nursing students, more and more high-quality classes have been established. Higher education curriculum construction has put forward higher requirements for nursing undergraduate teaching. Online and offline blended high-quality classes have become a mainstream form and the new normal of learning in the information age. However, at present many colleges and universities are in the initial stage of blended teaching, there are still a lot of problems, how to design and implement the blended teaching effectively is a huge challenge for teachers [1].

China’s Ministry of Education to create the “golden course” purpose is to make “students move, the classroom lively”, let students deeply involved in the classroom to cultivate the active learning and in-depth learning habits. However, differences in students’ initiative, participation in learning activities and differences in knowledge understanding in blended teaching make it difficult to achieve the “advanced” goal of “golden course” [2]. How to effectively connect and organically integrate online and offline teaching forms in nursing professional classroom has become an important issue facing nursing curriculum construction.

Obstetrics and gynecology (OB-GYN) nursing is a compulsory and core course for nursing students. Its teaching content is abstract, difficult to understand, professional and technical, emphasizing the combination of theoretical knowledge and clinical practice [3]. Teaching environment and teaching plan are the main factors affecting effective teaching of obstetrics and gynecology nursing, and are the effective means to achieve teaching objectives and the key to improve teaching quality [4].

In the literature, it has been reported that the traditional course of OB-GYN nursing focuses on the theoretical teaching and operation demonstration training, ignoring the cultivation of the comprehensive abilities such as self-learning, critical thinking, communication skills, practical application ability, and students used to passively accept knowledge [5]; However, simple online course learning lacks timely teaching interaction, and students have a poor sense of presence, which is not conducive to practical education of nursing specialty [6]. Therefore, it is particularly important to explore a more effective obstetrics and gynecology nursing teaching model.

In recent years, colleges and universities have actively implemented the blended teaching model to achieve personalized education in a variety of ways. In the field of nursing education, the blended teaching can stimulate students’ interest in learning, improve the learning effect and cultivate students’ comprehensive ability [7,8,9]. However, other studies have found that there is no statistical difference between the blended teaching group and the traditional teaching group in terms of grades, course satisfaction and independent learning readiness [10]. There are many studies on the application of blended teaching in the field of nursing education, but its design, implementation and evaluation methods are complex and diverse [11,12]. The simple combination of face-to-face learning and information technology cannot provide effective teaching and learning solutions, and there is still a lack of clear theoretical framework guidance.

Connectivist Learning was proposed by Siemens [13] as a means to understand and explore learning in a networked digital age. It explains how learning happens in the era of “Internet +” from a brand-new perspective. According to this theory, knowledge is a network phenomenon, and learning is the establishment of connections and the formation of networks, including neural networks, conceptual networks and external/social networks. The goal of learning is knowledge growth based on creation, that is, knowledge circulation. This theory is the first one to face the complexity of learning. It regards learning itself as a complex system, and “being” is an integral and distributed response to how elements are connected by the perceiver, and knowledge exists in the connection. Due to its forward-looking interpretation of human learning in the future, it has rapidly gained universal attention from the international community and become the highland and forefront of learning theory research [14]. Teaching interaction is the core of connectivist learning and the key to success, in order to apply connectivist learning to the education teaching practice, Wang et al. put forward a framework for interaction and cognitive engagement in connectivist learning. According to the cognitive participation from shallow to deep, the model divides the teaching interaction of connectionism learning into four levels: operation interaction, wayfinding interaction, sensemaking interaction and innovation interaction [15].

Guided by the framework for interaction and cognitive engagement in connectivist learning, this study constructed the online and offline blended teaching model of OB-GYN Nursing, in order to improve students’ autonomous learning, problem-solving skills, and the formation of critical thinking mode, achieve the goal of improving students’ comprehensive quality. At the same time, the study experience of nursing undergraduates in blended teaching was discussed by evaluating its implementation effect, and feedback was collected to provide reference for improving the application of blended teaching in nursing education. Hypothesized benefits were increased competency, self-directed learning level, and improved learning outcomes. Given that this is the first study to apply the framework for interaction and cognitive engagement in connectivist learning to nursing course, this study has the ability to identify potential benefits that could be used in future nursing education studies.

## 2. Methods

### 2.1. Study Design

The study utilized a randomized controlled trail design to examine nursing students’ comprehensive abilities after applying the blended teaching based on the framework for interaction and cognitive engagement in connectivist learning. The qualitative data was collected to examine the effects of the program.

### 2.2. Sample and Setting

This study was conducted between March and June 2021 in nursing department, Harbin Medical University. OB-GYN nursing is a mandatory class for junior students and there are six classes for nursing undergraduates. We randomly selected students from two classes and randomly allocated them to experimental group (n = 64) or control group (n = 59) by a sealed envelope system with two numbers (1-control; 2-experimental).

### 2.3. Ethical Statement

All participants signed the informed consent form, and this study was approved by the Institutional Review Board at Harbin Medical University, Daqing. All participants were told that they were free to withdraw from the study at any time and for any reason.

### 2.4. The Interaction and Cognitive Engagement-Based Blended Teaching Program

#### 2.4.1. Theoretical Framework

This study takes the framework for interaction and cognitive engagement in connectivist learning as the theoretical basis. The teaching model is constructed according to the four levels of interaction model. ① Operation interaction: the interaction between teachers, students and online resources can be realized in the online and offline teaching environment of blended teaching, so that students can establish contact and form feedback with teachers, classroom activities and online resources. ② Wayfinding interaction: Provide students with all kinds of information and instructions from teachers and teaching assistants before completing blended teaching, so that students can know how to conduct online self-study through online resources, and can clarify the teaching links, evaluation methods and rules of this course. ③ Sensemaking interaction: Through scientific organizational theory and practical teaching, students can reflect, summarize, share and make decisions. Through teaching activities, students can master and apply knowledge, so that they can make correct decisions in specific clinical situations. ④ Innovation interaction: On the basis of systematically mastering knowledge, knowledge points can be consolidated and integrated by creating and resynthesizing knowledge points, and horizontal and vertical knowledge can be closely linked to achieve the purpose of systematically mastering knowledge, and spiral knowledge consolidation and grid knowledge expansion and optimization can be carried out.

#### 2.4.2. The Blended Teaching Process Design

##### Teaching Objectives

The teaching objectives of this course focus on connecting with the cultivation of the core competence of nursing specialty, preparing for the nurse qualification examination and the needs of the learners. There is an emphasis on practicality and innovation with the development of the times. The teaching process pay more attention to the relationship between the vertical systematization of knowledge and the horizontal crosswise of knowledge so that students can master knowledge flexibly, moreover, it focuses on the cultivation of students’ critical thinking, promote students’ simple learning to in-depth thinking so that to reflect students’ participation in the blended teaching.

##### Teaching Contents

According to the teaching objectives, the new editions textbooks of Obstetrics and Gynecology, which are widely recognized in China Higher Education are used to make full preparation before class. Through collective lesson preparation with clinical obstetrics and gynecology professors and clinical teachers, the key and difficult points of each chapter were optimized and combined. In order to broaden students’ horizons, the new progress of obstetrics and gynecology and nursing was added in the teaching process.

**Figure 1:**
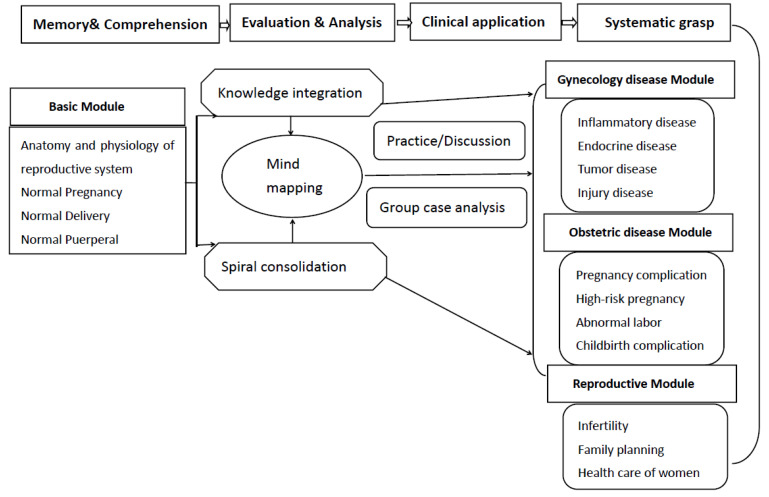
Divided modules of the course.

This course is divided into four progressive teaching modules to let students master the structured system knowledge, and the spiral teaching module division is shown in Figure 1.

##### Teaching Mode

Construct the “3(P) 2(R) 1(C) three stages” teaching mode.

3(P) includes **P**repare before class, as well as **P**resent and **P**roduce in class. For students, they need to complete the pre-class preparation and group discussion through the online platform (Prepare before class) and give report and complete discussion in groups according to the design of each class (**P**resent and **P**roduce in class).

2(R) includes **R**eview and **R**eflect after class. Students need to review and reflect what they have learned after class.

1(C) refers to Concept. Each student should form their own concept map of knowledge to achieve systematic mastery of theoretical knowledge of obstetrics and gynecology nursing.

Students are divided into several groups to finish the pre-class preparation and discussion. The group members have a clear division of task. The discussion topics include different diseases cases, critical thinking cases, and humanistic issues with ideological and political elements. Each student is responsible for their roles such as collecting data, sorting out data, making Power Point, drawing concept maps, and reporting offline classes, so they are deeply involved in the whole process of learning knowledge.

##### Teaching Organization Form

**Figure 2:**
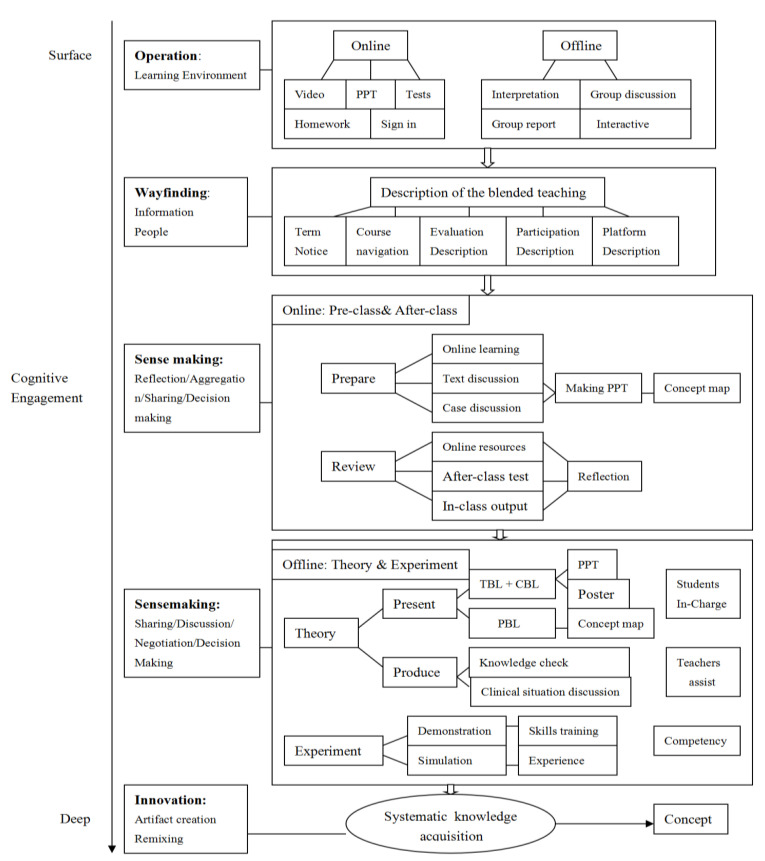
Framework for interaction and cognitive engagement in connectivist learning. Note: PPT-Power Point; TBL-Team-based learning; CBL-Case-based learning; PBL-Problem-based learning.

Through the establishment of teaching classes on the platform, the course knowledge videos, teaching Power Point, quizzes, check-in, online discussion, release and submission of homework can be connected before, during and after class and timely feedback on the relevant data of students’ participation level at each stage. The details of the platform were shown in Figure 2.

### 2.5. Control Group

To avoid the bias, the theoretical courses and clinical practice were administered to both groups by the same instructors. The students in this group received the usual teaching mode to complete the course tasks.

### 2.6. Instruments

#### 2.6.1. Final Course Exam

The examination paper is set by the instructor who independent of this study, the total score was 100 point which including the objective topic (single choice questions, multiple-choice questions) and the subjective topic (short-answer questions, the medical record analysis topic), the test time of 90 min, The two groups of students received the same examination paper, examination time and marking teacher.

#### 2.6.2. Competency Inventory for Nursing Students, CINS

The Chinese version of CINS scale was used to evaluate students’ professional core ability level, the scale has a total of 38 items, 6 dimensions which including basic biomedical science (5 items), the general clinical skills (6 items), critical thinking and reasoning (3 items), caring (5 items), ethics and accountability (14 items), lifelong learning (5 items). The total score ranges from 38 to 266, with higher scores indicating stronger core abilities. This scale was widely used among nursing students in China and has good reliability and validity [16]. The Cronbach’s alpha value in this study was found to be 0.80.

#### 2.6.3. Self-Directed Learning Instrument for Nursing Students, SDLINS

The Chinese SDLINS scale was used to compare the differences of self-directed learning ability between the two groups. The scale has a total of 60 items, which are composed of five dimensions (Awareness, Learning strategies, Learning activities, Evaluation, Interpersonal skills). The score range is 60–300 points, and the higher scores indicate the high level of independent learning ability. This scale was widely used among nursing students in China and has good reliability and validity [17]. The Cronbach’s alpha value in this study was found to be 0.81.

### 2.7. Data Collection

Before and after the course, questionnaires were sent to the Wechat group of the class in the form of links through the Platform of WenJuanXing to collect data of independent learning ability and core ability level of the two groups of students. Students were assured that their questionnaires would be anonymous and would not affect their grades.

### 2.8. Data Analyze

SPSS24.0 (IBM, Armonk, NY, USA) statistical software was used for data analysis. The descriptive categorical data were analyzed by using the number, percentage, median, minimum-maximum values, while the continuous data were analyzed using the arithmetic mean and standard deviation. Parametric test is used for normal distribution Paired T test was used to compare the score difference between the two groups. *p* < 0.05 was considered statistically significant.

## 3. Results

### 3.1. Comparison of Test Scores between Two Groups of Students

The theoretical test scores of the control group were 75.43 (5.22), and those of the intervention group were 77.25 (4.53). Paired T test was used to compare the scores of the two groups, and the difference was statistically significant (*t* = 2.57; *p* < 0.05).

### 3.2. Comparison of the CINS Scale Scores between Two Groups

There was no significant difference in the scores of CINS between the two groups before intervention (*p* > 0.05). After the course, the overall CINS score and scores of all dimensions in the experimental group were higher than those in the control group, with statistically significant differences between the two groups (*p* < 0.05), as shown in Table 1.

### 3.3. Comparison of the SDLINS Scale Scores between Two Groups

There was no significant difference in scores of self-directed learning ability between the two groups before intervention (*p* > 0.05). The total score and scores of all dimensions of self-directed learning ability of students in the intervention group were higher than those in the control group, with statistically significant differences between the two groups (*p* < 0.05), as shown in Table 2.

## 4. Discussion

The findings from this study show that the blended teaching method which based on the interaction and cognitive engagement facilitated a constructive opportunity for nursing students to developing a better performance in the OB-GYN learning and strengthen the general clinical skills, critical thinking and reasoning, caring ability, lifelong learning and self-directed learning.

With the increasingly mature network information technology widely applied in the field of education, the blended teaching mode based on flipped classroom has been widely used in nursing education. The core of flipped classroom is to move a lot of direct teaching out of the classroom by turning over the traditional classroom, thus freeing precious classroom time for meaningful deep learning. The key to the implementation of flipped classroom is not the pursuit of a stereotyped label, but the effective application of teaching mode according to different teaching situations.

Online teaching is an important part of blended teaching, and the connectivist learning theory is a new theoretical foundation for online education to adapt to the development of the times [18]. In the era of “Internet+”, the transformation of educational concept from “knowledge construction” to “knowledge production” reflects the transformation from passive to active knowledge treatment and from constructivism to connectivist [19].

Connectivist understands learning in a broader perspective and cultivates a wide range of learning and adaptability in learners in a dynamic and chaotic environment. Blended teaching is beneficial to cultivate learners’ learning ability. In the process of realizing the goal of “golden course”, it is more necessary for students to have good cognitive engagement [15].

The connectivist interaction model based on cognitive engagement describes the interaction rules and characteristics of learners in the learning environment of connectivist from the perspective of teaching interaction, and reveals the learning process of connectivist. According to the cognitive engagement from surface to deep, the model divides the teaching interaction of connectivist learning into four levels: “operational interaction”, “wayfinding interaction”, “sensemaking interaction” and “innovation interaction”. Lower-level interactions are the basis for higher-level interactions that extend the requirements of lower-level interactions. The 4-level interaction is a networked and nonlinear process, and shows strong recursion.

Based on the framework for interaction and cognitive engagement in connectivist learning, this course constructed a three-in-one, three-stage blended teaching model. By using the online and offline learning environment, it provided various opportunities to participate in the course activities, so as to achieve the purpose of “making students active and the class lively”. The teaching mode enabled students to carry out in-depth learning and consolidation of course knowledge in three stages. The experimental group students’ curriculum theory scores were higher than the control group, but also higher than the previous students’ scores, indicating that the blended teaching mode played an important role in consolidating knowledge points. The teaching content was divided into modules and the links and spiral consolidation between the four modules were emphasized in the details of knowledge points, so that students can achieve clinical application and systematic mastery on the basis of memory comprehension and evaluation analysis. This is also consistent with the essence of connectivist learning which is a spiral process of knowledge innovation and network expansion and optimization under the four types of interaction [20].

The results showed that the core competence and self-directed learning ability of the intervention group were significantly higher than those of the control group (*p* < 0.05). A number of investigations and studies have shown that nursing students, compared with theoretical reserve, lack of practical accumulation and low critical clinical thinking ability and autonomous learning ability [21,22]. Therefore, the blended teaching carried out in this study break through the traditional education mode of “subject-centered” and integrates knowledge teaching, ability cultivation and quality improvement, fully reflecting the organic combination of theory and practice, knowledge teaching and ability cultivation. Through the combination of “learned knowledge” and “knowledge that should be learned” with “how to learn knowledge”, students were constantly inspired to find blank spots in their own cognition and dig deeply into their own potential, so as to stimulate the improvement of nursing students’ professional core ability and independent learning ability [23]. This study runs through the idea of “students as the main body, teachers as the leading body”. The online course construction was carried out by the teacher team before class, and real clinical hot issues were added to the online materials and discussion cases in the form of network links, so as to maintain the innovation and modernity of the course content. This mode encouraged students to actively explore hot issues and controversial issues to improve the independent analysis, thinking, practice, questioning, creation and other abilities. By assigning pre-class tasks, students take the initiative to participate in exploration, and cultivate their ability to collect and process information, analyze and solve problems. According to the preview feedback from students, teacher designed class teaching content and applied a variety of teaching methods to inspire the student logical thinking ability and critical thinking ability, for example, in part of the teaching chapters; the industry-university-research collaborative project was integrated. It realizes the transformation from theoretical knowledge to practical application. Take the chapter of perineum care for example, when the students finished the class, they were invited to the perineum care company to guide the maternal, and during the nursing process, some students will find the needs of maternal and to do the research. It effectively improved students’ knowledge application ability and communication skills, and realized the effective combination of theory and practice.

The blended teaching has conducted the online discussion, project learning, peer mutual, case discussion and report and other activities [24]. The online discussions and autonomous learning mentioned the highest frequency during the students’ interviews. Contemporary college students are more inclined to the characteristics of meaning learning, by raising questions to trigger thinking and discussion, by completing the project learning way to promote cooperative learning which is conducive to improve students’ independent learning ability [25]. Online self-learning in blended teaching is a test of students’ self-management ability and active learning ability, which will directly affect the involvement and participation of online learning, and the higher involvement rate, the better the learning effect [15]. Therefore, in order to achieve good teaching effect in blended teaching, the quality of online teaching resources should be improved, so as to strengthen students’ pre-class preparation and independent learning ability, and form deep participation in class and after class.

As a new teaching mode, there are still many aspects needs to be improved, including the setting of learning content, the diversification of learning forms, the adjustment of course difficulty and the supervision of learning effects. In the study some students reported that as the course progressed, their learning enthusiasm declined, which may be related to the longer course length and the decrease of students’ interest. Therefore, teachers need to pay attention to the change of students’ learning enthusiasm at any time, and take measures to improve students’ learning enthusiasm. Critz and Knight [26] proposed that students’ problems should be detected and intervened early, and teaching methods should be improved in time to ensure students’ enthusiasm in learning. Schlairet [27] proposed that teachers should find appropriate ways to manage students’ learning expectation and process. At the same time, due to the increase of students’ learning autonomy in blended teaching, how to carry out process assessment, accurately reflect students’ learning effect, and how to supervise students’ learning process are all problems that need to be considered in future studies.

## 5. Limitations

Although this study was a RCT design there are some limitations to our study. First, the study was conducted with nursing students in one university; the generalizability of the results should be interpreted with caution. Second, only the effects of the competence of nursing students and the self-directed learning ability of nursing students were examined, future studies might consider analyzing the effects on other outcomes.

## 6. Conclusions

In this study, the blended teaching mode based on the framework for interaction and cognitive engagement in connectivist learning was applied to obstetrics and gynecology nursing course, which achieved good teaching effect, effectively improved the core ability of nursing students, and cultivated the self-directed learning ability of nursing students. This preliminary application results show that the teaching mode is recognized by students and the cognitive participation and teaching satisfaction are high, in future studies, the suitable, efficient, modern teaching mode can be applied to other nursing courses to strengthen the discipline integration and improve the comprehensive ability of nursing students.

## Figures and Tables

**Table 1:** Comparison of the CINS scale scores between two groups (point, M(SD)).

Item	Experimental Group (n = 64)	Control Group (n = 59)	*t*	*p*
Clinical biomedical science	24.25 (2.31)	21.91 (2.17)	5.47	<0.01
General clinical skills	33.07 (1.94)	31.26 (1.88)	5.02	<0.01
Critical thinking and reasoning	13.41 (1.19)	11.57 (1.63)	7.40	<0.01
Caring	25.01 (1.85)	22.91 (1.96)	5.71	<0.01
Ethics and accountability	76.17 (1.23)	73.83 (2.26)	6.17	<0.01
Lifelong Learning	27.68 (1.91)	26.07 (2.15)	4.78	<0.01
Total score	199.62 (4.53)	187.51 (7.55)	11.11	<0.01

**Table 2:** Comparison of the SDLINS scale scores between two groups (point, M(SD)).

Item	Experimental Group (n = 64)	Control Group (n = 59)	*t*	*p*
Awareness	49.03 (1.17)	47.98 (1.75)	3.83	<0.01
Learning activities	50.00 (1.63)	48.10 (2.17)	6.12	<0.01
Learning strategies	47.60 (2.30)	49.28 (1.78)	4.33	<0.01
Evaluation	49.50 (1.93)	48.41 (1.94)	2.76	<0.01
Interpersonal skills	50.05 (1.66)	48.97 (2.23)	3.25	<0.01
Total score	247.86 (3.75)	241.07 (5.47)	7.57	<0.01

## Data Availability

The data presented in this study are available on request from the author. The data are not publicly available due to ethical restrictions.
